# An initiative on digital nephrology: the Kidney Imageomics Project

**DOI:** 10.1093/nsr/nwaf034

**Published:** 2025-02-21

**Authors:** Fangxu Zhou, Zehua Li, Haifeng Li, Yao Lu, Linjia Cheng, Ying Zhang, Zichen Wang, Jing Nie, Heping Cheng, Bin Dong, Lei Ma, Li Yang

**Affiliations:** Renal Division, Peking University Institute of Nephrology, Peking University First Hospital, China; Key Laboratory of Renal Disease—Ministry of Health of China, Key Laboratory of CKD Prevention and Treatment (Peking University)—Ministry of Education of China, Peking University First Hospital, China; Research Units of Diagnosis and Treatment of Immune‐Mediated Kidney Diseases—Chinese Academy of Medical Sciences, Peking University First Hospital, China; National Biomedical Imaging Center, College of Future Technology, Peking University, China; Renal Division, Peking University Institute of Nephrology, Peking University First Hospital, China; Key Laboratory of Renal Disease—Ministry of Health of China, Key Laboratory of CKD Prevention and Treatment (Peking University)—Ministry of Education of China, Peking University First Hospital, China; Research Units of Diagnosis and Treatment of Immune‐Mediated Kidney Diseases—Chinese Academy of Medical Sciences, Peking University First Hospital, China; Beijing International Center for Mathematical Research and the New Cornerstone Science Laboratory, Peking University, China; Renal Division, Peking University Institute of Nephrology, Peking University First Hospital, China; Key Laboratory of Renal Disease—Ministry of Health of China, Key Laboratory of CKD Prevention and Treatment (Peking University)—Ministry of Education of China, Peking University First Hospital, China; Research Units of Diagnosis and Treatment of Immune‐Mediated Kidney Diseases—Chinese Academy of Medical Sciences, Peking University First Hospital, China; Renal Division, Peking University Institute of Nephrology, Peking University First Hospital, China; Key Laboratory of Renal Disease—Ministry of Health of China, Key Laboratory of CKD Prevention and Treatment (Peking University)—Ministry of Education of China, Peking University First Hospital, China; Research Units of Diagnosis and Treatment of Immune‐Mediated Kidney Diseases—Chinese Academy of Medical Sciences, Peking University First Hospital, China; Academy for Advanced Interdisciplinary Studies, Peking University, China; National Biomedical Imaging Center, College of Future Technology, Peking University, China; National Biomedical Imaging Center, College of Future Technology, Peking University, China; Renal Division, Peking University Institute of Nephrology, Peking University First Hospital, China; Key Laboratory of Renal Disease—Ministry of Health of China, Key Laboratory of CKD Prevention and Treatment (Peking University)—Ministry of Education of China, Peking University First Hospital, China; Research Units of Diagnosis and Treatment of Immune‐Mediated Kidney Diseases—Chinese Academy of Medical Sciences, Peking University First Hospital, China; National Biomedical Imaging Center, College of Future Technology, Peking University, China; State Key Laboratory of Membrane Biology, Peking University, China; Institute of Molecular Medicine, College of Future Technology, Peking University, China; Peking-Tsinghua Center for Life Sciences, Peking University, China; National Biomedical Imaging Center, College of Future Technology, Peking University, China; Beijing International Center for Mathematical Research and the New Cornerstone Science Laboratory, Peking University, China; Center for Machine Learning Research, Peking University, China; National Biomedical Imaging Center, College of Future Technology, Peking University, China; Renal Division, Peking University Institute of Nephrology, Peking University First Hospital, China; Key Laboratory of Renal Disease—Ministry of Health of China, Key Laboratory of CKD Prevention and Treatment (Peking University)—Ministry of Education of China, Peking University First Hospital, China; Research Units of Diagnosis and Treatment of Immune‐Mediated Kidney Diseases—Chinese Academy of Medical Sciences, Peking University First Hospital, China

The kidney's complex structure and functions are essential for homeostasis. Each human kidney contains one million nephrons surrounded by a vast network of peritubular capillaries that support oxygen and nutrients, and an interstitial compartment that produces hormones such as erythropoietin and calcitriol. Due to its complex structure and functions, the kidney is particularly vulnerable to metabolic disorders, immune dysregulation, ischemia, infections and toxins. These insults can lead to the development of chronic kidney disease (CKD) and acute kidney injury (AKI) [1]. CKD is the third leading cause of diminished global life expectancy, posing a significant public health challenge worldwide [2]. Limitations in early diagnostics and effective treatments contribute to AKI incidence rates of 20%–50% among high-risk inpatients, with associated mortality rates exceeding 25% and 50% of the patients progressing to CKD [3].

Improving kidney disease outcomes requires innovative strategies for early diagnosis, timely prevention and effective treatment. Recent advances in omics technologies—such as genomics, proteomics and metabolomics—have propelled both clinical and basic research, providing insights into the molecular and biochemical underpinnings of kidney diseases. To address the need for precision nephrology, international initiatives such as Neptune (https://www.neptune-study.org/), Trident (https://www.med.upenn.edu/trident/) and CureGN (https://pnrconsortium.org/publications-grants/curegn), have been

launched to advance precision nephrology and improve patient management. Among these efforts, the Kidney Precision Medicine Project (KPMP, *https://www.kpmp.org/*) stands out by using spatial and single-cell omics to build a comprehensive human atlas of kidney injury, enabling insights into cellular and molecular mechanisms and advancing precision nephrology [4].

Kidney injury involves complex processes across regions and renal cell types, spanning scales from the macroscopic down to the microscopic. These processes include injury and repair mechanisms driven by functional, structural and molecular alterations, with injury etiology manifesting in a range of spatial patterns—whether diffuse or focal, global or segmental. This spatial heterogeneity and cross-scale complexity poses challenges in unraveling kidney injury mechanisms at a whole-organ level [1]. Recent advances in imaging technologies have begun to bridge these gaps, offering tools to study kidney diseases with unprecedented detail across multiple scales. Techniques such as synchrotron radiation X-ray (SRX) and cryo-MOST have enabled high-resolution mapping of whole-kidney structures [5,6], while photoacoustic imaging provides critical insights into the renal microcirculatory system [7]. Multiphoton microscopy complements these approaches by enabling real-time *in vivo* fluorescence imaging of kidney processes at subcellular resolution [8], linking structural and functional observations. Building on these advances, the emerging

field of imageomics offers an integrated approach to bridge complex biological layers, enabling holistic comprehension of kidney injury across macroscopic to molecular levels with unprecedented clarity.

## IMAGEOMICS: BRIDGING MULTIMODAL AND CROSS-SCALE INFORMATION

Omics systematically quantifies and integrates various features of a domain within one sample to provide a comprehensive characterization. Imageomics, as omics for imaging, leverages multimodal imaging technologies and advanced computational methods to systematically capture and combine all features across multiple scales. This interdisciplinary approach complements molecular omics, bridging the gap between molecular intricacies and whole-organ physiology, thereby providing a cohesive framework for understanding biological complexity [9]. Given the complementary strengths and limitations of different imaging modalities [5], integrating multiple techniques is essential for advancing kidney disease diagnosis and research.

China's National Biomedical Imaging Center (NBIC) (https://nbic.pku.edu.cn) is positioned to lead in advancing imageomics. As a leading institution providing unparalleled imaging capabilities that cover a spatiotemporal scale of 10 orders of magnitude, NBIC is dedicated to systematically developing all the technological capabilities necessary for truly grasping complex life systems. Equipped with three major imaging platforms that encompass macro-, meso- and microscopic scales, NBIC integrates advanced technologies such as high-field MRI, multiphoton optical microscopy, synchrotron radiation bioimaging beamlines, multi-beam scanning electron microscopy and high-resolution mass spectrometry imaging microscopes. Recent breakthroughs in imaging technologies have also been applied to kidney research, such as using multiphoton microscopy to visualize IgA deposition in the kidneys of BALB/c nude mice [8], and utilizing SRX to reconstruct the 3D structure of human kidneys at micron resolution [5]. Beyond providing state-of-the-art imaging for functional, structural and molecular-level observations, NBIC features a ‘full-scale data integration system’, equipped with 1800 + TFLOPS (trillion floating-point operations per second) of CPU, 3000 + TFLOPS of GPU computing power and a 40 + PB distributed storage system. This infrastructure is optimized for high-throughput biomedical-imaging-data processing, supporting advanced data fusion and easy access for large-scale, multimodal analysis. Additionally, its software system, designed for 3D visualization, multi-channel interaction and smart automation, helps us to comprehensively understand life and disease mechanisms at molecular, cellular and organismal levels. Together, these resources provide essential support for advancing kidney imageomics and exploring its potential in precision nephrology.

## THE KIDNEY IMAGEOMICS PROJECT

The Kidney Imageomics Project (KIP), initiated by the imageomics consortium organized by the renal division of Peking University First Hospital (PKUFH) and NBIC, is funded by the National Natural Science Foundation of China (NSFC), National Key R&D Program of China and NBIC. This project is set to last for at least 10 years and encourages international collaboration. The KIP aims to advance precision nephrology by creating a multimodal and cross-scale imageomics digital atlas of the entire kidney. In contrast to the above-mentioned initiatives that focus on specific aspects of kidney disease predominantly by molecular omics and pathology, KIP uniquely integrates multimodal, multiscale imaging to construct a comprehensive digital atlas of kidney injury.

**Figure 1.**
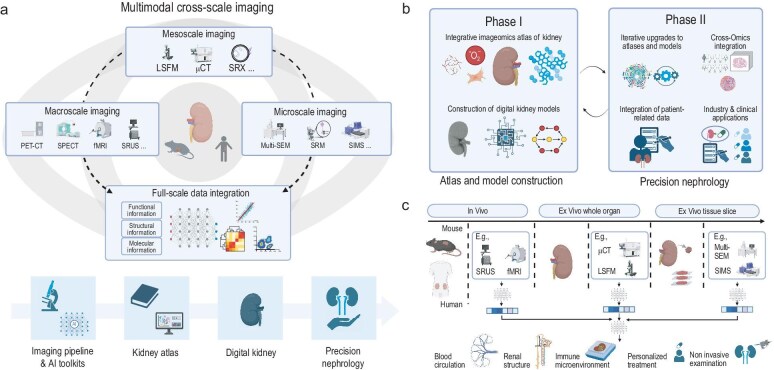
(a) Roadmap of KIP. The Kidney Imageomics Project (KIP) aims to advance precision nephrology by creating a comprehensive multimodal, cross-scale imageomics digital atlas of the entire kidney. It is supported by the National Biomedical Imaging Center (NBIC), which provides macro-, meso- and microscopic scale imaging, along with a full-scale integration system. KIP begins by establishing a high-quality multimodal imaging workflow and AI analysis toolkits to construct a multimodal, multiscale imageomics atlas. This atlas will enable systematic disease characterization, decoding of disease mechanisms and support renal simulations and drug response predictions through the construction of digital kidney models. Ultimately, by translational clinical applications and advancing new technologies, KIP aims to advance the transformation of kidney disease diagnosis and treatment, driving the evolution of precision medicine. PET-CT, positron emission tomography–computed tomography; SPECT, single photon emission computed tomography; fMRI, functional magnetic resonance imaging; SRUS, super-resolution ultrasound; LSFM, light sheet fluorescence microscopy; uCT, micro-computed tomography; SRX, synchrotron radiation X-ray; SEM, scanning electron microscopy; SRM, super-resolution microscopy; SIMS, secondary ion mass spectrometry; AI, artificial intelligence. (b) Phased goals of KIP. In the initial phase, sub-atlases focusing on functional, structural and molecular dimensions will be developed to delineate the distinct compartments of the kidney. By integrating functional, structural and molecular imaging modalities across various scales, KIP aims to construct a multimodal cross-scale imageomics atlas of kidney injury. Furthermore, based on these atlas data, a multiscale, multimodal modeling framework for *in silico* experimentation will be established, allowing simulations to predict kidney function, injury progression and responses to interventions within a five-year timeframe. In subsequent phases, the atlas will undergo continuous optimization and updates, enhancing interpretability, prediction accuracy and model precision through collaboration with large patient cohorts. The ultimate aim is to advance precision medicine and personalized healthcare for kidney diseases. Looking ahead, KIP plans to further integrate multi-omics data, expanding the diversity and resolution of the atlas for broader biomedical insights. (c) Integrated technical framework of KIP. The KIP technical framework involves macro-, meso- and microscopic imaging of animal and human kidneys *in vivo*, intact *ex vivo* and sectioned *ex vivo*, facilitated by NBIC imaging platforms. Functional, structural and molecular data are collected and transmitted for multimodal, cross-scale data integration, supporting the development of a digital kidney atlas. This integrated data set enables detailed modeling of kidney blood flow, 3D structures and immune microenvironment, providing valuable insights for translational research, precision therapy and non-invasive 3D kidney pathology.

To achieve our aims, the project will follow this technical roadmap (Fig. 1a):

- Establishing compatible imaging pipelines and integrative analysis toolkits to create a multimodal imageomics atlas for animal and human kidneys.
- Conducting systematic atlas interpretation, spanning both functional and structural imaging at tissue, cellular and molecular levels.
- Developing an organ-level digital model of kidney function simulation and prediction.

Each step contributes to precision nephrology (Fig. 1b):

- Imaging Technologies and Artificial Intelligence (AI) Tool Development: This foundational step establishes non-invasive, dynamic and real-time multimodal diagnostic capabilities for kidney diseases. It also facilitates the design of innovative instruments and analytical tools, forming the basis for a comprehensive diagnostic framework.
- Multimodal Imageomics Atlases: These atlases integrate imaging data with molecular and clinical information to uncover novel mechanisms and therapeutic targets, identify diagnostic biomarkers and enhance disease detection and stratification.
- Digital Kidney Models: By simulating disease progression and predicting patient-specific therapeutic responses, these models enable the development of personalized treatment strategies and optimized interventions.

Data generated by the KIP will be systematically organized and openly shared, fostering global collaboration and enabling free exploration of kidney-related research.

## KIDNEY ATLAS CONSTRUCTION

### Data acquisition

The primary challenge of kidney imageomics is creating an automated technical pipeline that balances multimodal compatibility with the imaging quality of each modality. Sample preparation and the imaging pipeline for each modality have been thoroughly analyzed and optimized for compatibility. *Functional imaging:* Techniques like photoacoustic imaging, functional magnetic resonance imaging (fMRI) and non-invasive molecular imaging at a resolution of 10–100 μm provide a dynamic dissection of various renal functional units (nephron and microcirculation) and detect perfusion, oxygenation and metabolism of the whole kidney. *Structural imaging:* SRX enables panoramic 3D multiscale imaging of intact human kidneys without contrast toxicity, supporting AI-driven virtual histology [5]. For microscopic structural imaging, super-resolution microscopy (SRM) tracks subcellular structures and biomolecules with improved spatial-temporal resolution [10]. Volumetric electron microscopy enables 3D nanoscale reconstructions for precise quantification of intracellular organization and organelle compartmentalization [11]. *Molecular imaging:* Spatial molecular imaging omics now profiles thousands of genes or proteins with subcellular resolution, even in formalin-fixed tissue, enhancing compatibility with tissue pathology [5]. Tissue clearing techniques enable multiplexed protein co-localization in 3D intact kidneys, supporting subsequent pathological and electron microscopy analysis [5].

Leveraging these technologies, KIP developed an integrated imageomics workflow to address compatibility and imaging-quality challenges (Fig. 1c). After non-invasive *in vivo* functional imaging, paraformaldehyde perfusion fixation is performed for the subsequent structural imaging such as computed tomography (CT) scans and pathology, at a resolution of micrometers or sub-micrometers. To bridge the structural and molecular modalities, tissue clearing technology is utilized to visualize the nephron structure in 3D. Additionally, a deep learning model is set up to infer cell type information from structural imaging, allowing for detailed images of cell states or signaling networks. This enables pairing CT/pathology and spatial omics data from the same renal slice using formalin-fixed paraffin-embedded (FFPE), a method for preserving tissue samples, based spatial omics strategies such as Xenium and PhenoCycler Fusion (PCF) [5]. Based on multimodal compatibility, KIP has optimized imaging quality of each modality. Given the kidney's fine structure and diverse cell types, which demand high resolution and multiplexing capabilities, KIP prioritizes developing novel materials, staining techniques and device optimization. These efforts aim for quantifiable, dynamic, panoramic high-resolution imaging with satisfactory contrast and safety.

### Data integration

KIP takes advantage of the latest advancements in AI to create a comprehensive digital kidney atlas by integrating multimodal imaging data. This requires a data-processing framework capable of tackling challenges like variations in data quality, large-scale data sets and the inherent heterogeneity across imaging modalities. *Variability in data quality* from diverse imaging devices, along with the sheer volume of multimodal data, complicates processing and analysis workflows. KIP utilizes the NBIC's full-scale data integration system to mitigate these difficulties by streamlining data collection, automating quality control and minimizing manual intervention to optimize workflow efficiency. High-resolution panoramic imaging of the whole kidney generates data on the order of petabytes (PB). *Large-scale data sets* are supported by the system's substantial storage capacity and computational power, enabling advanced deep learning for tasks such as feature extraction and complex data preprocessing. We also emphasize the need for strict data security protocols to ensure that all data usage complies with privacy regulations. *Data heterogeneity across imaging modalities*, such as inconsistencies in image intensity, resolution and tissue deformation, poses another challenge. KIP addresses this by applying AI-driven segmentation and image registration networks to align data from identical samples, enabling cohesive assessment of kidneys [12]. For non-identical samples, self-supervised learning can align heterogeneous data within the latent space for subsequent analysis [13].

Building on this data processing framework, *supervised and weakly supervised AI models* are used to enhance kidney structure segmentation precision and enable detailed quantitative analyses of disease-related morphological changes [14]. Precise segmentation supports rigorous evaluation of structural alterations related to disease. Furthermore, *self-supervised multimodal AI models*, including transformer-based architectures, are used for data integration across modalities by synthesizing imaging data with other modalities, enhancing KIP's diagnostic and prognostic capabilities [15,16]. Additionally, *foundation models* pre-trained on large-scale data sets reveal complex links between phenotypes and kidney diseases, potentially offering deeper insights into pathophysiological mechanisms [17]. To ensure AI safety, a wide range of diverse data sets are employed, rigorous validation processes are put in place, and continuous error monitoring is conducted to prevent bias and inaccuracies. By systematically integrating these advanced AI technologies, KIP addresses multimodal data processing challenges and establishes a reliable and comprehensive digital kidney atlas, essential for advancing our understanding of renal health and disease.

### Digital kidney

In life sciences, predictive modeling is increasingly leveraging Digital Twins (DTs) to simulate biological interactions spanning from the cellular, organ levels all the way to the individual level [18,19]. These simulations help anticipate disease progression and evaluate interventions, offering a powerful tool for personalized medicine. However, developing a Digital Kidney in KIP is challenging due to the kidney's multiscale complexity. Additionally, achieving model generalizability across patients is difficult, as kidney injuries vary across individuals and disease stages in space and time. Model validation and interpretability are essential, and AI-driven predictions should be biologically plausible and clinically actionable.

To accurately model the kidney, KIP will gather comprehensive 3D structural and functional imaging. This extensive data collection is crucial for developing a robust digital kidney model to simulate complex functions over time. The digital kidney aims to integrate patient-specific data (clinical records, imaging, time-course measurements) with a template kidney model to create a personalized one [18]. This personalized model forecasts disease progression and outcomes, guiding tailored treatment. By simulating interventions at molecular, cellular and tissue levels, it optimizes treatment plans with real-time adjustments. Integrating diverse data sets enhances model accuracy, and iterative refinement adjusts both data collection methods and model parameters for clinical relevance. Ultimately, these efforts with regard to the digital kidney will support virtual clinical trials, guiding personalized treatment and improving kidney-disease patient outcomes.

## HUMAN TRANSLATIONAL APPLICATION

Human translational applications of the imageomics digital atlas are vital for precision medicine. However, establishing a human kidney atlas presents many challenges, with much progress yet to be made. The larger volume and complex relationships of the human kidney complicate multiplex labeling and necessitate a more careful balance between resolution and field of view (FOV) in imaging. Furthermore, limited availability of fresh samples due to ethical concerns poses challenges in database building. To address these, KIP will focus on optimizing technical instruments, enhancing imaging parameters, developing novel probes, advancing multimodal AI algorithms, refining FFPE compatibility and accumulating human data through multi-center cohorts with ethical approval, consent obtainment, privacy protection and data supervision. Considering the challenges of complete kidney imaging, KIP aims for two distinct atlases of the complete human kidney by utilizing a multimodal *in vivo* imaging cohort alongside *ex vivo* structural and molecular imaging.

To advance the atlas for precision nephrology, KIP will prioritize cross-species transfer learning [20], enhance atlas interpretation, increase throughput with automation and integrate clinical data. The transition of novel *in vivo* imaging technologies from model organisms to clinical applications promises innovative diagnostics quite soon, thereby speeding up clinical translation. Using advanced AI architectures, KIP's cross-species atlas based on transfer learning may reveal new disease biomarkers, subtypes and trajectories, deepening our understanding of underlying mechanisms. Fully automated data workflows will support efficient, large-scale data accumulation. Leveraging these data sets, novel deep-learning frameworks, such as biomedical foundational models, will enable nuanced interpretations of complex multimodal data sets, aiding in smarter diagnostics and elucidating intricate pathophysiological mechanisms.

Incorporating predictive modeling as a key tool, the human kidney atlas can simulate disease progression, therapeutic responses and individual patient outcomes. A digital kidney model helps clinicians optimize medication selection and dosage based on patient's kidney structure, disease stage and molecular profile. Furthermore, by correlating imaging phenotypes with clinical features, KIP seeks to foster a paradigm shift in personalized medicine with more precise multimodal models, improving patient stratification and targeted interventions, and ultimately enhancing patient care and outcomes.

## KIP AS AN EXPANDABLE FRAMEWORK

The boundaries between various omics disciplines are increasingly blurred. As resolution and FOV improve, the fusion of high-dimensional pathomics and subcellular imageomics becomes feasible. Furthermore, the expansion of data scale and human translation driven by AI pave the way for a profound integration with phenomics [21]. The KIP framework ensures integration with various omics to enrich the information domain and extend it to molecular and whole-organ levels. Thus, in pursuit of precision medicine and individualized treatment strategies for kidney disorders, KIP will remain an expandable and flexible framework that incorporates additional modalities and welcomes collaborations with colleagues across diverse disciplines, including clinical physicians, pathophysiologists, imaging specialists, omics researchers and artificial intelligence experts, in an effort to accelerate the realization of precision nephrology.
